# Detecting signatures of positive selection associated with musical aptitude in the human genome

**DOI:** 10.1038/srep21198

**Published:** 2016-02-16

**Authors:** Xuanyao Liu, Chakravarthi Kanduri, Jaana Oikkonen, Kai Karma, Pirre Raijas, Liisa Ukkola-Vuoti, Yik-Ying Teo, Irma Järvelä

**Affiliations:** 1 NUS Graduate School for Integrative Science and Engineering, National University of Singapore, Singapore 117456, Singapore; 2 Department of Medical Genetics, University of Helsinki, P.O. Box 63, 00014 University of Helsinki, Finland; 3 DocMus Department, University of the Arts Helsinki, P.O. Box 86, 00251 Helsinki, Finland; 4 Joensuu Conservatory, Rantakatu 31, 80100 Joensuu, Finland; 5 Saw Swee Hock School of Public Health, National University of Singapore, Singapore 117597, Singapore

## Abstract

Abilities related to musical aptitude appear to have a long history in human evolution. To elucidate the molecular and evolutionary background of musical aptitude, we compared genome-wide genotyping data (641 K SNPs) of 148 Finnish individuals characterized for musical aptitude. We assigned signatures of positive selection in a case-control setting using three selection methods: haploPS, XP-EHH and F_ST_. Gene ontology classification revealed that the positive selection regions contained genes affecting inner-ear development. Additionally, literature survey has shown that several of the identified genes were known to be involved in auditory perception (e.g. *GPR98, USH2A*), cognition and memory (e.g. *GRIN2B*, *IL1A*, *IL1B*, *RAPGEF5*), reward mechanisms (*RGS9*), and song perception and production of songbirds (e.g. *FOXP1*, *RGS9*, *GPR98*, *GRIN2B)*. Interestingly, genes related to inner-ear development and cognition were also detected in a previous genome-wide association study of musical aptitude. However, the candidate genes detected in this study were not reported earlier in studies of musical abilities. Identification of genes related to language development (*FOXP1* and *VLDLR*) support the popular hypothesis that music and language share a common genetic and evolutionary background. The findings are consistent with the evolutionary conservation of genes related to auditory processes in other species and provide first empirical evidence for signatures of positive selection for abilities that contribute to musical aptitude.

Human abilities to appreciate and practice music are well preserved in evolution^1^,^2^,^3^. The discovery of 40,000-year old flutes in recent archaeological excavations has suggested that recognizable musical behaviours must have existed several years before that^4^. An inter-species evolutionary background of musical behaviour has also been suggested^3^. What are the reasons for the existence of music and musical abilities throughout human history?Are there any genetic alleles that transmit musical practices through generations?

The majority of human-specific traits, including advanced cognitive abilities, are thought to have evolved as a result of positive selection^5^. Favourable alleles proliferate in the gene pool showing high allele frequencies associated with the beneficial trait, whereas damaging alleles that might cause harmful effects to the trait may disappear from the gene pool. Alleles under positive selection increase in prevalence in a population, leaving “signatures” or patterns of genetic variation on the DNA sequence^6^. Therefore, the identification of genes/regions under positive selection would provide novel insights into the molecular genetic background of human-specific traits^7^. Studies on the positive selection signals in human evolution have identified genes underlying wide spectrum of biological functions like dietary adaptation, physical appearance, behaviour, sensory systems and cognitive abilities^7^. For example, genes associated with cognition-related traits like visual perception, language, speech and song-learning (e.g. opsin genes and *FOXP2*) have been found to be under positive selection in human evolution^8^,^9^.

Musical aptitude is fundamental and necessary for many music related activities including for example creativity in music, music performance and dance. Here, we have applied genomic and bioinformatics approaches to assign the regions of positive selection associated with musical aptitude in the human genome. Musical aptitude relies upon the ability to perceive and understand intensity, pitch, timbre and tone duration, as well as the rhythm and structure they form in music. In genetic terms, it represents a complex cognitive trait in humans. Previously, we have performed a genome-wide linkage and association study with musical aptitude, which identified genes related to inner-ear development and cognition as being crucial for musical aptitude^10^. Here, we used three metrics, two haplotype-based methods haploPS^11^ and XP-EHH^12^, and the allele frequency-based method F_ST_^13^ to identify subject-specific, genome-wide differential positive selection signals in musical aptitude.

## Results

### Candidate selection regions identified by haploPS

**Figure 1:**
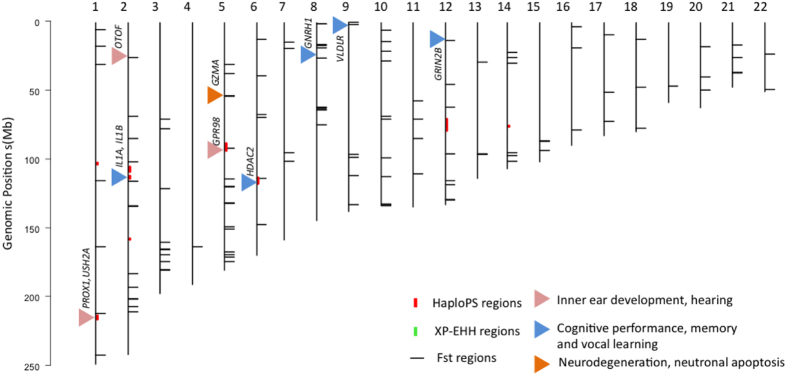
Selection regions identified by haploPS, XP-EHH and Fst. The red bars denote case specific regions identified by haploPS, green bars denotes the regions identified by XP-EHH in case. Fst selection regions are denoted as black lines. Genes with functions that are relevant to music aptitude such as hearing, cognitive performance and neurodegenerative functions are marked by triangles.

With 74 cases and 74 controls (Table 1, Supplementary Table S1), haploPS identified 46 candidate selection regions in the cases at 5% significance threshold and 86 selection regions in the controls at 10% significance threshold. Case specific signals are those identified at 5% significance level in the cases but are still absent at 10% significance threshold in the controls. Twelve case specific selection regions were identified (Fig. 1 and Table 2).

The best two regions were found at chromosome 12 (12q15–q21.2, haploPS P-value = 0.0053; 12q21.2, haploPS P-value = 0.0077). Both regions contained many brain-related but also other genes (Table 2). In the other resulted regions, we also identified genes with brain functions but also genes with numerous other effects, as most of the resulting haplotypes extended several megabase pairs long and included several genes. For example, the third best region at 2q12 (haploPS P-value = 0.0115) extended nearly 3 Mb and included *NCK2* that is linked to the growth of neuronal cells via Robo-Slit-pathway^14^.

Among the rest of the 9 case specific regions, we identified a few genes that are functionally related to brain functions and hearing. The region at 2q13 containing *IL1A* and *IL1B* is found to be under selection uniquely in the cases (haploPS P-value = 0.0343). Both genes were found to affect cognition, where *IL1B* was associated with baseline cognitive performance among older adults, and IL1B was associated with working memory^15^,^16^. A case-specific signal on chromosome 17 (haploPS P-value = 0.0362) contained *RGS9* gene, which belongs to the regulator of G-protein signaling (RGS) gene family^17^. The longer splice variant of RGS9 (RGS9-2) is specifically expressed in the striatum, which is involved in controlling movement, motivation, mood and reward and is known to be affected by music^18^,^19^. There is evidence for a specific functional interaction between RGS9-2 and striatal D2-like dopamine receptors^20^. Music-induced reward is further elucidated by the release of dopamine in the mesolimbic striatum when experiencing peak emotional arousal during music listening^21^.

A case specific signal on chromosome 5 (5q14.3) encapsulating the *GPR98* (haploPS P-value = 0.0378), also known *VLGR1*, has been found to associate with Usher syndrome type 2C, whose major symptoms include sensorineural deafness. *GPR98* is required for normal development of auditory hair bundles^22^. Furthermore, *GPR98* was identified by Nam *et al.* to be among the 58 neurological genes evolving under positive selection specifically in songbird lineage (zebra finch), when compared to the chicken lineage, and was differentially expressed in song control nuclei of zebra finch brain suggesting a function in birdsong^23^. The region on chromosome 1q32.3–41 contains *USH2A* (haploPS P-value = 0.0391), which encodes *usherin*, and is responsible for most of the Usher syndrome cases that are characterized by deafness and blindness. *USH2A* is found to be essential in the normal development of cochlear hair cells that may in turn affect hearing sensitivity^24^.

### Positive selection regions identified by XP-EHH

XP-EHH identified 9 selection regions in the cases, using the controls as the reference population. (Fig. 1 and Table 3) Within top signals, *RAPGEF5*, a member of the Rap guanine nucleotide exchange factors, was found. Rap guanine nucleotide exchange factors members are found to be enriched in the brain and act as regulators of the interplay between Rap proteins and Ras protein, which are implicated in region-specific learning and memory functions^25^,^26^. The exact function of *RAPGEF5* is not clearly elucidated; however, another member of the same family, *RAPGEF4,* was shown to play an important role in memory retrieval^25^,^26^. Among genes within a significant selection region on chromosome 8 encapsulates the *GNRH1* gene, which belongs to the hypothalamic gonadotropin-releasing hormone (GnRH) family. The GnRH family has been linked to hippocampal sex differences in the zebra finch and implies that the difference may contribute to sex differences in song learning and memory^27^. Most of the other genes had unknown function.

### Candidate selection regions identified by F_ST_

A total of 128 significant regions were identified from the F_ST_ analysis (Fig. 1, Supplementary Table S2). Of them, the top 20 signals are shown in Table 4. The most significant region detected by F_ST_ metric at 5q11.2 covers *GZMA*, *GPX8*, *MCIDAS*, *CCNO* and *DHX29* genes (regional F_ST_ = 0.1393). (Fig. 1 and Table 4). The function of *GZMA* was implicated in the neurodegeneration mechanisms^28^ and was down-regulated after listening to classical music in experienced listeners in our recent study^29^. *GRIN2B* on chromosome 12 encodes a subunit of N-methyl-D-aspartate (NMDA) receptor, a subtype of ionotropic glutamate-gated ion channel whose role is known in learning and brain plasticity. Polymorphisms in *GRIN2B* were found significantly associated with cognitive performance; brain structure in the temporal lobe^30^,^31^. *GRIN2B* is among the singing regulated transcripts, expressed in the striatum of finch^32^,^33^.

Interestingly, *FOXP1* on chromosome 3p13 (regional Fst 0.07361) and *VLDLR* chromosome 9p24.2 (regional F_ST_ = 0.0646), which are well-known genes involved in human language development and cognitive disorders were also identified^34^,^35^. The *VLDLR* gene is a direct target of FOXP2, which is a transcription factor identified to be critical for learned vocal-motor behaviour such as singing and speech^36^. *VLDLR,* together with *FOXP2* were found to be members of singing-regulated gene networks unique to the song-specialized basal ganglia subregion, striatopallidal area X in the zebra finch. The protein product of *VLDLR*, very-low-density lipoprotein receptor (Vldlr) is a member of the Reelin pathway, which was identified to be underlying learned vocalization^37^. Overexpression of *VLDLR* also results in hyperactivity and impaired memory in rats^38^.

*OTOF* identified in a candidate selection region detected by F_ST_ metric (regional F_ST_ = 0.063) on chromosome 2p23, has been substantially studied for its role in hearing and multiple mutations in this gene have been reported to be associated with hearing loss^39^. *OTOF* encodes a transmembrane protein otoferlin, which was demonstrated to function in the exocytosis at the auditory inner hair cell ribbon synapse.

### Gene ontology classification

Gene ontology classification (GeneTrail2, Hypergeometric distribution test, Benjamini and Yekutieli false discovery rate adjustment method^40^) of the genes falling within the candidate selection regions identified by HaploPS, XP-EHH and F_ST_ window statistics revealed the enrichment of genes responsible for inner ear development (*DICER1, FGF20, CUX1, SPARC, KIF3A, TGFB3, LGR5, GPR98, PAX8, COL11A1, USH2A,* and *PROX1*) (p-value 0.002) and cellular component assembly involved in morphogenesis of different organs (*DICER1, PCM1, TBC1D7, KIF3A, CCNO, MCIDAS, IQCB1, FOXP1, HDAC2, BMP10, TTLL5, BBS10, RSPH4A,* and *PROX1*) (p-value 0.002).

## Discussion

In this empirical study, three metrics (haploPS, XP-EHH and F_ST_) were used to identify positive selection regions underlying musical aptitude in the human genome. The definition of the phenotype was based on pattern recognition^41^ combined with sensory capacities to detect pitch and duration^10^. With the musical aptitude phenotypes, we partitioned the study subjects into cases (higher musical aptitude scores) and controls (lower musical aptitude scores). Regions showing differential evidence of positive selection between cases and controls were subsequently identified as candidate genomic regions of positive selection associated with COMB scores (Fig. 1).

Biological interpretation of selection regions is difficult. On one hand, the function of the majority of the genes identified in selection regions is unknown. On the other hand, haplotype based methods identify long-range haplotypes that underpin natural selection signals. The large regions may extend over multiple genes, and we are not able to underpin the actual selected gene. We have listed in the results the genes whose functions were well-studied and related to brain functions, hearing and are expressed in songbirds’ brain. However, we would like to emphasize that the genes mentioned may not represent the actual gene under selection, and more functional studies on the genes are needed to elucidate the functional importance of the selection regions. As musical aptitude is a complex cognitive trait, there is a possibility that these areas may contain genes related to other music-related phenotypes like general cognitive capacities.

Several genes affecting inner ear development (*DICER1, FGF20, CUX1, SPARC, KIF3A, TGFB3, LGR5, GPR98, PAX8, COL11A1, USH2A,* and *PROX1*) were identified to be under positive selection as observed in gene ontology analysis. The finding is plausible as the structure and function of the auditory system is very similar in modern humans and the first primates, suggesting high evolutionary conservation of auditory perception among species^42^,^43^,^44^.

*FOXP2* has been implicated in human speech and language^45^ and it has been the target of positive selection during recent human evolution^46^. Another candidate gene *VLDLR* is known to be a direct target gene of human *FOXP2*, which is a transcription factor identified to be critical for learned vocal-motor behaviour such as singing and speech^36^,^46^. A number of studies have reported an overlap between the neural and behavioural resources of language and music^47^,^48^, thus suggesting a common evolutionary background of language and musical abilities. Our results support these findings.

Interestingly, the candidate regions contained several genes that are known to be involved in the song perception and production processes of songbirds (e.g., *FOXP1, GPR98, RGS9, GRIN2B, VDLDR)*, which further suggest a possible evolutionary conservation of biological processes related to musical aptitude. For example, several of the candidate genes detected by F_ST_ were reported to be responsible for song learning^32^ and singing^33^ in songbirds. The findings are consistent with the convergent evolution of genes related to auditory processes and communication in other species^43^. In total, ~5% of the identified candidate genes were known to be responsible for vocal learning and singing in songbirds^32^,^33^. Particularly, one of the identified candidate genes *GPR98*, is known to be expressed in the song control nuclei of vocalizing songbird (zebra finch), and has been found to be under positive selection in songbird lineage^32^, thus making it a plausible candidate gene for the evolutionary advantage of human musical aptitude. The identified selection region containing *GPR98* extended more than 4 Mb on chromosome 5 by haploPS (Table 2). Part of the 4 Mb region overlaps with a region identified with F_ST_ although the overlapping region does not cover *GPR98*. Further studies are needed to investigate whether the common selection region detected by the two methods is linked to musical aptitude. Moreover, *GPR98* and *USH2A* genes are not only associated with hearing but also with vision. Synesthesia is a condition where one sensory stimulus can cause a sensation in other sensory stimuli. Synesthesia has been reported in musicians who can hear sounds in different colors^48^. This finding suggests a common molecular background of sensory perception skills.

Common region between haploPS and F_ST_ was found at chromosome 6 (haploPS P-value = 0.0280; F_ST_ = 0.06616) where *MARCKS*, *LOC285758*, *FLJ34503* and *HDAC2* genes were found from the shared region. Histone deacetylase 2 encoded by *HDAC2* represses genes that are crucial for learning and memory by histone deacetylation and affects cognition by suppressing synaptic excitation and enhancing inhibitory synaptic function in hippocampal neurons^49^,^50^.

The reward value of music has been suggested as a reason for the possible positive selection for musical aptitudes in human evolution, where increased dopamine secretion induced by listening to music has been justified to play a role^18^,^19^,^20^,^21^. Recent genomic studies of music perception and performance have further provided evidence for genes related to dopaminergic pathways affected by listening and performing music^29^,^51^. Among these genes, the expression of *RGS2* was increased after listening to music^29^ and *RGS2* has also been implicated in song learning and singing in songbirds^52^. In this study, another member of the RGS-gene family, *RGS9,* was identified in a selection region using haploPS. Interestingly, both *RGS2* and *RGS9* are expressed in striatum where the strongest reward value of musical stimuli has been demonstrated^53^. Dorsal component of striatal regions accompanied with prefrontal cortex is activated by expectations and the fulfilment of expectations leads to dopamine release in the striatum^54^. In fact, the phenotype used in this study defines the ability to form expectations in music structure^55^,^56^,^57^,^58^. Thus, the RGS proteins represent novel candidate genes underlying the evolution of music, whose association with musical aptitude needs further verification.

The study population is an isolated population^59^, and longer stretches of haplotypes are expected than those observed in cosmopolitan populations. We cannot exclude the possibility that the observed haplotypes are characteristic to the Finnish population. HaploPS searches for signals of positive selection by first searching for long haplotypes at each haplotype frequency across the genome. Uncharacteristically long haplotypes are identified from the distribution of haplotype length and the distribution of the number of SNPs on the haplotypes to represent positive selection regions. The method has been applied to isolated populations, such as the aboriginal populations from Southeast Asia, to detect positive selection signals^60^. Simulations have also shown that in the presence of strong bottlenecks in European populations, the false positive rate of haploPS is still well maintained^11^. For example, in the extreme case where inbreeding coefficient equals 0.3, the false positive rate of haploPS is 6.7%. XP-EHH is also a haplotype-based selection method that requires a reference population to detect selection signals in the target population. In this study, using the same population controls as the reference population eliminates the potential bias that may be caused by different demographic histories of reference and target populations (Figure S1). Significant XP-EHH scores are expected to be associated with musical aptitude. For methodological differences, the resulting signals from XP-EHH were shorter than those of haploPS and thus, the regions included only a few genes. Though both methods are haplotype-based, not many overlapping signals are expected as demonstrated in previous studies^11^. Indeed, we did not observe common signals between the results of the two methods. Although study material was partially shared between this study and a recent genome-wide linkage and association study on musical aptitude^10^, the identified genomic regions did not overlap substantially. However, the genes related to the inner-ear development and cognitive processes were identified in both the studies.

The significance thresholds of the selection methods haploPS, XP-EHH and F_ST_ are the verified cutoff values and used in previous studies implementing the methods^11^,^12^. Application of selection detection methods in case-control settings help linking candidate case-specific selection regions to phenotypes under selection, and thus facilitate biological interpretations of positive selection regions. Our selected genes discussed above represent top statistically significant genes that have a link to the phenotype used in this study. At the same time, we do acknowledge the other genes that we did not discuss in the manuscript. Musical aptitude is a polygenic trait, and our study has identified multiple selection regions related to musical aptitude, using haplotype-based and frequency-based selection methods. Future direction includes applying reliable selection methods that are designed for detecting polygenic selection, once such methods are available^61^.

We and others^3^,^10^,^62^,^63^ have proposed that musical aptitude is necessary for the development of music culture. As genetic evolution is much slower than cultural evolution, we hypothesize that the genetic variants associated with musical aptitude have a pivotal role in the development of music culture. This is supported by studies on song learning behaviour in songbirds that have taught that the evolution of song culture is the result of a multigenerational process where the song is developed by vertical transmission in a species-specific fashion suggesting genetic constraints^64^. This emphasizes the importance of selection of parental singing skills and their genetic background in evolution. In parallel, musical aptitude in humans needs early exposure to music to be developed and that is mediated through teaching, imitation and other forms of social learning^3^,^6^. Our study serves as a starting point to identify molecules and pathways that contribute to the evolution of musical traits. Obviously, further studies are required to assess the crosstalk between genetic variants and the aforementioned environmental exposure of music, at different ages, in larger study materials and in different populations.

## Materials and Methods

### Ethics statement

The Ethical Committee of the Helsinki University Central Hospital approved the study and written informed consent was obtained from all participants or their parents. The methods were carried out in accordance with the approved guidelines.

### Participants and phenotypes

We surveyed 283 unrelated Finnish subjects (age range 13–86; 40% males) where the musical aptitude of each subject was assessed using three music tests: the auditory structuring ability test (Karma Music Test, KMT)^41^,^55^ and Carl Seashore’s subtests of pitch (SP) and time discrimination (ST)^56^. The KMT is based on pattern recognition where small, abstract sound patterns are repeated to form hierarchic structures^41^. KMT measures the recognition of melodic contour, grouping, relational pitch processing, and gestalt principles^55^. The KMT is based on pattern recognition in a group of tones that are heard three times. The fourth time the pattern is either the same or changed that the listeners are asked to recognize^55^. Pattern recognition in KMT is related to cued associations in music structure and sound sequence learning also present in speech and motor sequence learning^56^. An example of three items belonging to KMT can be listened on http://www.hi.helsinki.fi/music/english/samples.htm. Seashore’s test for pitch (SP) measures the ability to detect the difference in pitch of two sequential tones, whereas Seashore’s test for time (ST) measures the ability to detect small differences in tone duration. The auditory structuring ability can be considered conceptionally unrelated although somewhat correlated to these sensory capacities^10^,^41^. To get a more comprehensive phenotype for musical aptitude, a combined test score (COMB; range 75–150) was introduced to represent the measured musical aptitude as a single variable, and it was computed as the sum of the three test scores after suitable adjustment to bring the scores to the same scale^10^. We clarify here that the definition of a fundamental human capability such as musical aptitude cannot depend on changing cultural definitions of music. The auditory structuring ability contains the ability to identify temporal aspect in time (detecting sound patterns in time) that is related to pattern recognition in many other fields like in poetry (comprising language and speech), visual Morse code and in several types of sport that would also be highly musical activities.

With the COMB phenotypes, we partitioned the study subjects into cases (higher musical aptitude scores) and controls (lower musical aptitude scores). Regions showing differential evidence of positive selection between cases and controls are subsequently identified as candidate genomic regions of positive selection associated with musical aptitude. Genes affecting COMB scores were identified to be underlying the evolution of musical aptitude in the population. To accomplish this, a linear regression was fitted between the COMB score and age of samples. The distribution of the residual is roughly normal (Shapiro-Wilk normality test p-value = 1.56 × 10^−4^). We defined samples with residuals smaller than −10 as controls with low COMB scores and those with residual larger than 11 as cases with high COMB scores. A total of 74 controls representing low musical aptitude and 74 cases representing high musical aptitude were obtained for downstream analysis. The COMB scores range from 75 to 150. As a result of assigning cases and controls, controls have their scores below 117.25 and cases above 125 (Table 1). PCA analysis has shown the cases and controls are homogenous to the Finnish population (Supplementary Figure S1). Variations in music test scores suggest that the predisposing alleles may have been targeted for selection.

### Genotyping and quality control criteria

Genomic DNA was extracted from peripheral blood using the phenol-chloroform method. Genotyping was performed on the Illumina HumanOmniExpress 12 1.0 V SNP chip (Illumina Inc., San Diego, CA, USA). Genotype calls were performed with GenomeStudio. Quality control of the genotype data was performed using the PLINK software^65^. Of the 290 samples in the original dataset, we removed a sample from subsequent analyses if the sample exhibited any of the following: (i) more than 5% missing genotypes across all the SNPs; (ii) excess heterozygosity, which is indicative of sample contamination; (iii) a high level of identity-by-state genotypes, which is indicative of relatedness or duplicates in which case we retained only the sample in each pairing with the higher call rate. SNPs were excluded if they: (i) remained monomorphic across all samples; (ii) exhibited a minor allele frequency <5%; (iii) exhibited more than 5% missing genotypes; and (v) exhibited gross deviation from Hardy-Weinberg equilibrium as defined by *P*_HWE_ < 10 ^7^. This yielded a combined dataset of 641,417 SNPs across 283 samples.

### Haplotype phasing

The phases of the haplotypes for 283 samples were estimated from the genotype data with the software *Beagle* version 3.3.2^66^. This was performed with all 283 samples within a single batch run.

### Positive selection analysis

We aimed to find differential positive selection signals that are present in the cases, but absent in the controls. To locate signature of positive selection, we performed genome-wide scans of selection signals using three metrics: haplotype-based methods haploPS^11^ and XP-EHH^12^, and the allele frequency based method F_ST_^13^.

HaploPS is used to identify selection signals in both cases and controls, and case specific signals can then be identified from the comparison. HaploPS searches for uncharacteristically long haplotypes when compared to the rest of the haplotypes in the genome at a given frequency^11^. At each given focal SNP, haploPS quantifies the length of longest haplotype formed around this SNP at a pre-defined frequency based on the genetic distance spanned (in cM) and the number of SNPs on the haplotype. An empirical significance score was calculated by quantifying the relative length of each haplotype at a given frequency after scaling by the total number of regions found across the whole genome. A positive selection region was identified if the empirical measure of the region is smaller than the significance threshold of 0.05. This procedure was performed within the population across haplotype frequencies that range from 0.05 to 0.95 at a step-size of 0.05. Genomic regions identified at multiple haplotype frequencies in the same region were represented by the significant haplotype with the highest frequency in the region. Differential selection signals were defined as regions that are significant at 5% significance level in the cases, but still fail at 10% threshold in the controls.

The cross-population extended haplotype homozygosity (XP-EHH) used the control data as the reference population and cases as the target such that the XP-EHH results will represent selection signals that are specific to the cases. The XP-EHH compares the extended haplotype homozygosity between two populations at each focal SNP^12^. XP-EHH analyses a common set of SNPs that are present in both populations, and SNPs located within 1 Mb of a focal SNP were used to calculate the XP-EHH score, defined as the logarithm of the ratio of the area under the EHH curves for the target population to that of the reference population. The genome-wide raw XP-EHH statistics were standardized to a distribution with zero mean and unit variance. SNPs in the top 0.1% are taken as significant SNPs. Significant regions are identified by combining SNPs of significant XP-EHH scores that are less than 200 kb apart. If two SNPs both have significant XP-EHH scores and are less than 200 kb apart, then the two SNPs forms a region. More SNPs are added to the region if they are significant are less than 200 kb away from the region.

We used F_ST_ as another indicator of differential positive selection between the cases and the controls. F_ST_ quantifies the allele frequency differences between cases and controls, and regions with contiguous stretches of large F_ST_ values are expected to harbour positive selection signals. In more detail, the Weir & Hill’s F_ST_^13^ was calculated between cases and controls for the 641417 SNPs with the expression F_ST_ = (MSP−MSG)/(MSP + |(n_c −1)*MSG), where 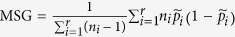 and 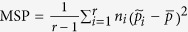. n_i_ is the sample size of each population. After calculating the Weir and Hill unbiased F_ST_ for all SNPs in the dataset, we divided the whole genome into 200 kb non-overlapping windows and computed a window statistic (mean F_ST_ of top 3 SNPs). Regions undergoing positive selection are expected to have large population frequency difference between cases and controls. In each window, windows with less than 5 SNPs were dropped from the analysis, and the average of the top three F_ST_ values was taken as the test statistics of the window. Based on the window statistics, we define the windows with statistics in the top 1% of the distribution as significant regions of positive selection. Regions showing differential evidence of positive selection between cases and controls were subsequently identified as candidate genomic regions of positive selection associated with musical aptitude.

### Gene ontology classification

The selection regions were annotated using human genome build hg19. We used Genetrail2 (http://genetrail2.bioinf.uni-sb.de/) to categorize the biological functions of the candidate genes identified by all the three methods (haploPS, XP-EHH and F_ST_). A hypergeometric distribution test with a false discovery rate of 0.005 was used for the overrepresentation analysis. The clustering of gene families in a single candidate locus may lead to the inflated significance of some genes. To avoid this problem, overrepresented gene ontology terms were reported only if the enriched genes were detected across multiple candidate genetic loci.

## Additional Information

**How to cite this article**: Liu, X. *et al.* Detecting signatures of positive selection associated with musical aptitude in the human genome. *Sci. Rep.* **6**, 21198; doi: 10.1038/srep21198 (2016).

## Supplementary Material

Supplementary Information

## Figures and Tables

**Table 1:** Characteristics of the participants.

		Cases	Controls
COMB	Min	125	76
	Max	148	117.25
	Mean	137.41	100.30
Age	Min	18	13
	Max	70	86
	Mean	44.41	44.99
Gender	Males	38	27
	Females	36	47

**Table 2:** Case specific selection signals identified by haploPS.

Chr	Start position	End position	Genetic Distance(cM)	Adjustd haploPS P-value	Estimated frequency	Genes
1	213862164	216608268	3.269	0.0391	0.05	LINC00538, PROX1, SMYD2, PTPN14, CENPF, KCNK2, KCTD3, USH2A
1	102959259	103763252	0.356	0.0292	0.4	COL11A1
2	158193407	158291539	0.176	0.0197	0.9	CYTIP
2	112409842	113989236	1.360	0.0342	0.1	ANAPC1, MERTK, TMEM87B, FBLN7, ZC3H8, ZC3H6, RGPD8, TTL, POLR1B, CHCHD5, FLJ42351, SLC20A1, CKAP2L, IL1A, IL1B, IL37, IL36G, IL36A, IL36B, IL36RN, IL1F10, IL1RN, PSD4, PAX8
2	105982753	109034995	3.846	0.0115	0.05	FHL2, LOC285000, NCK2, C2orf40, UXS1, PLGLA, RGPD3, ST6GAL2, LOC729121, RGPD4, SLC5A7, SULT1C3, SULT1C2, SULT1C2P1, SULT1C4
5	175136782	175202064	0.310	0.0373	0.9	
5	89001243	93774632	3.206	0.0378	0.05	MIR3660, CETN3, MBLAC2, POLR3G, LYSMD3, GPR98, LUCAT1, ARRDC3, ARRDC3, NR2F1, NR2F1, MIR548AO, FAM172A, MIR2277, POU5F2, KIAA0825
6	113555945	117792143	2.872	0.0280	0.05	MARCKS, LOC285758, FLJ34503, HDAC2, HS3ST5, FRK, TPI1P3, NT5DC1, COL10A1, TSPYL4, TSPYL1, DSE, FAM26F, TRAPPC3L, FAM26E, FAM26D, RWDD1, RSPH4A, ZUFSP, KPNA5, FAM162B, GPRC6A, RFX6, VGLL2, ROS1
12	76280316	79305654	4.981	0.0077	0.05	PHLDA1, NAP1L1, BBS10, OSBPL8, ZDHHC17, CSRP2, E2F7, NAV3, SYT1
12	71139664	76116463	4.088	0.0053	0.05	PTPRR, TSPAN8, LGR5, ZFC3H1, THAP2, TMEM19, RAB21, TBC1D15, MRS2P2, TPH2, TRHDE, TRHDE, LOC100507377, ATXN7L3B, KCNC2, CAPS2, GLIPR1L1, GLIPR1L2, GLIPR1, KRR1
14	76064624	76439634	0.246	0.0440	0.6	FLVCR2, C14orf1, TTLL5, TGFB3
17	63154324	63353913	0.343	0.0362	0.55	RGS9

**Table 3:** Selection signals detected by XP-EHH in cases, using controls as the reference population.

Chr	Start position	End position	peakID	Peak XP-EHH score	Genes
1	63361822	63567957	1	5.1412	
2	204833578	205051247	7	4.9179	
4	18332045	18553713	4	5.1927	
7	22010092	22222507	6	6.4088	RAPGEF5
8	25194779	25406521	8	4.9747	DOCK5, GNRH1, KCTD9, CDCA2
10	114980485	115180485	9	4.9126	
12	21360603	21569820	2	5.2474	SLCO1B1, SLCO1A2, IAPP
16	86307223	86525343	3	6.2781	LOC146513, LINC00917, FENDRR
21	17658842	17875056	5	4.9894	LINC00478

**Table 4:** Top 20 selection regions identified by Fst.

Chr	Start	End	No. of snp	window.fst	Genes
5	54392373	54563480	38	0.139	GZMA,CDC20B,GPX8,MIR449A,MIR449B,MIR449C,MCIDAS,CCNO,DHX29
5	54187813	54379426	57	0.136	ESM1,GZMK
5	120328270	120524720	40	0.120	
22	49484533	49683905	142	0.094	
18	13271319	13471199	60	0.088	LDLRAD4, MIR5190
10	6657775	6857219	101	0.087	LINC00707
1	18090975	18289252	76	0.087	ACTL8
12	118885039	119083566	54	0.086	
13	96590060	96777428	19	0.085	UGGT2, HS6ST3
12	115616511	115816442	81	0.085	
14	30395066	30593866	48	0.084	PRKD1
8	75207653	75405213	42	0.083	JPH1,GDAP1
5	174481433	174678058	87	0.082	
12	129547345	129745507	129	0.082	TMEM132D
3	165751527	165942272	34	0.081	
14	101743391	101942768	63	0.081	
20	49960636	50160035	71	0.080	NFATC2,MIR3194
9	133250275	133442778	73	0.080	ASS1
12	13927062	14125734	78	0.079	GRIN2B
11	111037249	111233450	61	0.079	C11orf53,C11orf92,C11orf93,MIR4491,POU2AF1
