# Supplementary material for: Detecting signatures of positive selection associated with musical aptitude in the human genome
Source: Sci Rep. 2016 Feb 16;6:21198. doi: 10.1038/srep21198 (PMC4754774; doi:10.1038/srep21198)
Supplement: Supplementary Information [file srep21198-s1.pdf]

Supplementary info

Detecting signatures of positive selection associated with musical aptitude in the human genome

Xuanyao Liu [1,5],Chakravarthi Kanduri [2], Jaana Oikkonen [2], KaI Karma [3], Pirre Raijas [4], Liisa Ukkola-Vuoti [2], Yik-Ying Teo [1,5], Irma Järvelä [2*]

[1]NUS Graduate School for Integrative Science and Engineering, National University of Singapore, Singapore 117456, Singapore

[2]Department of Medical Genetics, University of Helsinki, P.O. Box 63, 00014 University of Helsinki, Finland

[3]DocMus Department, University of the Arts Helsinki, P.O. Box 86, 00251 Helsinki, Finland

[4]Joensuu Conservatory, Rantakatu 31, 80100 Joensuu, Finland

[5]Saw Swee Hock School of Public Health, National University of Singapore, Singapore 117597, Singapore

Table S1. Characteristics of the participants

ID	GENDER	COMB	SP	KMT	AGE	Case/Control
**174**	1	142,25	37	47	48	Case
**727**	1	141,25	37	47	53	Case
**100**	2	129	28	46	65	Case
**106**	1	142	40	46	27	Case
**108**	2	132,25	37	45	48	Case
**172**	2	141	40	48	34	Case
**712**	2	147,75	39	50	36	Case
**33**	2	143,75	39	48	31	Case
**82**	2	138	36	45	55	Case
**534**	2	139,75	39	48	37	Case
**847**	1	136,5	34	49	40	Case
**137**	1	137,5	34	49	54	Case
**143**	1	133,5	34	47	53	Case
**187**	1	135,5	34	46	53	Case
**190**	1	141,75	39	47	33	Case
**225**	1	133,25	37	46	47	Case
**228**	2	133,25	37	45	43	Case
**238**	1	137	36	48	49	Case
**866**	2	148	40	49	27	Case
**255**	2	142	40	48	49	Case
**256**	2	128	40	48	58	Case
**260**	1	138,25	37	49	59	Case
**861**	1	148	40	49	46	Case
**280**	2	133,25	37	44	39	Case
**859**	1	129,5	38	44	51	Case
**329**	2	137,5	38	46	28	Case
**874**	1	142	40	48	40	Case
**380**	2	129	36	48	58	Case
**675**	2	143	40	49	40	Case
**679**	1	135,25	37	43	40	Case
**432**	1	137,5	38	48	52	Case
**480**	1	139	36	48	48	Case
**686**	1	144	40	49	23	Case
**606**	2	125,5	30	47	70	Case
**689**	1	142,5	38	48	57	Case
**412**	2	134	36	46	45	Case
**493**	1	129,5	34	38	51	Case

**495**	1	133,5	34	44	47	Case
**507**	1	131,5	34	43	60	Case
**695**	2	127	36	36	62	Case
**525**	1	148	40	50	54	Case
**516**	2	134,25	37	48	56	Case
**528**	2	144	40	48	50	Case
**570**	2	137,25	37	47	31	Case
**536**	2	136,75	39	45	36	Case
**574**	1	130,5	30	46	58	Case
**599**	2	137,25	37	47	40	Case
**611**	2	125	32	48	67	Case
**622**	1	128	28	46	59	Case
**623**	1	140,5	38	45	23	Case
**626**	1	129,25	29	47	55	Case
**633**	2	133,75	39	45	44	Case
**641**	1	134,75	39	48	36	Case
**642**	2	137,75	39	47	39	Case
**647**	1	127	32	45	69	Case
**649**	1	142	40	47	30	Case
**655**	1	144,75	39	49	34	Case
**660**	2	133	36	45	58	Case
**691**	2	143	40	49	51	Case
**705**	2	143,75	39	48	18	Case
**728**	2	140,75	39	48	21	Case
**729**	1	143,5	38	50	21	Case
**738**	1	138,25	37	48	42	Case
**765**	2	144,5	38	49	34	Case
**767**	1	148	40	49	18	Case
**768**	2	143,75	39	50	49	Case
**770**	1	131,25	33	47	45	Case
**790**	1	143	40	49	31	Case
**794**	1	141,5	38	50	24	Case
**852**	2	138,25	37	48	36	Case
**863**	2	135,5	38	44	54	Case
**414**	1	137	40	48	48	Case
**694**	2	129,25	37	44	67	Case
**800**	2	140,5	38	48	32	Case
**200**	2	80,75	23	27	85	Control
**715**	2	114,5	22	41	21	Control
**113**	2	103,5	30	28	57	Control
**714**	2	111	20	44	30	Control
**48**	2	96	20	34	50	Control

**129**	2	104	20	35	59	Control
**134**	1	100,25	25	28	60	Control
**857**	2	100,25	29	34	49	Control
**745**	2	100,75	27	35	68	Control
**38**	1	95,25	29	34	72	Control
**9**	1	104,5	26	30	19	Control
**5**	2	105,75	31	29	27	Control
**313**	2	99,25	29	32	60	Control
**363**	1	95,75	27	25	31	Control
**150**	2	89,5	26	25	51	Control
**661**	2	117,25	25	46	20	Control
**204**	1	104,25	25	37	52	Control
**212**	2	83,5	22	25	45	Control
**222**	1	92,25	25	25	61	Control
**234**	1	105	20	42	57	Control
**229**	1	96	20	35	86	Control
**232**	2	104	20	42	58	Control
**248**	2	91,25	25	25	52	Control
**267**	1	111,25	33	27	41	Control
**871**	2	104,75	23	34	53	Control
**275**	2	93	20	30	56	Control
**276**	1	99,75	23	34	41	Control
**293**	2	103,25	29	25	46	Control
**296**	2	107	20	44	42	Control
**324**	2	103,25	25	27	30	Control
**875**	2	106,75	27	36	27	Control
**387**	1	103,25	25	32	56	Control
**339**	1	102,75	27	30	67	Control
**518**	1	107,75	27	35	49	Control
**669**	2	96,75	27	25	81	Control
**384**	2	91	20	37	59	Control
**674**	2	93,5	22	28	42	Control
**676**	2	93	20	36	70	Control
**430**	2	110,25	25	47	28	Control
**464**	1	110,5	22	47	37	Control
**476**	1	103	28	26	58	Control
**685**	2	106,5	30	27	30	Control
**548**	2	99,75	23	33	36	Control
**544**	1	108,25	29	29	37	Control
**690**	2	105,75	27	38	36	Control
**583**	2	108,25	29	25	48	Control
**569**	1	106	28	34	32	Control

**396**	2	107	24	40	50	Control
**573**	2	101	20	35	52	Control
**590**	2	114,75	31	33	30	Control
**605**	1	84	20	34	27	Control
**607**	1	76	20	26	83	Control
**613**	2	96,5	26	28	54	Control
**617**	2	109,75	23	42	44	Control
**630**	1	98,75	23	36	23	Control
**643**	2	106,5	26	36	26	Control
**648**	2	93	20	34	18	Control
**653**	2	103,25	29	25	13	Control
**663**	2	92	24	25	24	Control
**681**	1	98	20	39	59	Control
**698**	2	89	20	25	37	Control
**700**	1	99,25	25	37	50	Control
**719**	2	94	28	25	68	Control
**732**	2	106,25	29	41	45	Control
**744**	2	112,5	30	34	28	Control
**778**	2	113	32	32	30	Control
**785**	1	98,5	26	27	31	Control
**788**	1	103,75	23	35	42	Control
**797**	1	101,5	22	35	43	Control
**826**	2	76	20	26	13	Control
**832**	2	108,75	27	48	15	Control
**837**	1	85	20	25	54	Control
**838**	2	83,75	23	27	65	Control
**844**	1	100,5	30	25	33	Control

Table S2. Positive selection region associated with musical aptitude detected by Fst.

Chr	Start	End	No. of SNPs	window.fst	Genes
**1**	6212843	6411024	47	0.065	CHD5,RPL22,RNF207,ICMT,LINC00337,HES3,GPR153,ACOT7,
**1**	18090975	18289252	76	0.087	ACTL8,
**1**	31278481	31471702	68	0.076	SDC3,PUM1,SNORD103B,SNORD103A,SNORD85,
**1**	115672664	115870310	75	0.064	NGF,
**1**	163645519	163845420	44	0.066	
**1**	212295571	212474288	44	0.072	PPP2R5A,
**1**	242599000	242798972	78	0.063	PLD5,
**2**	26522041	26720434	46	0.063	GPR113,EPT1,DRC1,OTOF,
**2**	69004457	69201894	91	0.066	ARHGAP25,BMP10,GKN2,GKN1,
**2**	85124209	85321746	33	0.068	TMSB10,KCMF1,
**2**	102160250	102359923	42	0.076	MAP4K4,
**2**	116362694	116551962	31	0.073	DPP10,
**2**	133838236	134034022	47	0.064	NCKAP5,
**2**	134242471	134441766	71	0.067	NCKAP5,
**2**	183151764	183350379	45	0.071	PDE1A,
**2**	193046220	193238289	29	0.063	TMEFF2,
**2**	201656457	201854175	24	0.064	AOX2P,BZW1,CLK1,PPIL3,NIF3L1,ORC2,FAM126B,
**2**	202149696	202348303	29	0.065	CASP8,ALS2CR12,TRAK2,STRADB,
**2**	207470884	207665145	59	0.077	ADAM23,LOC200726,DYTN,MDH1B,FASTKD2,MIR3130,MIR3130,
**2**	211151774	211341349	39	0.071	MYL1,LANCL1,
**3**	71212990	71412654	60	0.074	FOXP1,
**3**	78064944	78256536	19	0.066	
**3**	121481801	121670405	58	0.065	IQCB1,EAF2,SLC15A2,
**3**	160499192	160696867	34	0.064	PPM1L,

**3**	165337109	165505177	32	0.073	BCHE,
**3**	165751527	165942272	34	0.081	
**3**	169679950	169861358	39	0.067	LOC100128164,SEC62,GPR160,PHC3,
**3**	174767848	174966522	61	0.064	NAALADL2,NAALADL2,
**3**	180544880	180737641	20	0.065	FXR1,DNAJC19,
**3**	180749249	180932119	19	0.070	
**4**	163808442	164007992	32	0.066	
**5**	31518912	31717468	90	0.068	DROSHA,C5orf22,
**5**	37984257	38176521	72	0.072	
**5**	54187813	54379426	57	0.136	ESM1,GZMK,
**5**	54392373	54563480	38	0.139	GZMA,CDC20B,GPX8,MIR449A,MIR449B,MIR449C,MCIDAS,CCNO,DHX29,
**5**	92040221	92236350	53	0.072	
**5**	114733752	114928381	48	0.076	FEM1C,TMED7,TICAM2,
**5**	120123994	120318488	31	0.069	
**5**	120328270	120524720	40	0.120	
**5**	131951996	132151071	44	0.064	RAD50,IL13,IL4,KIF3A,CCNI2,SEPT8,SOWAHA,
**5**	132355147	132550830	36	0.071	ZCCHC10,HSPA4,FSTL4,
**5**	149276198	149467663	66	0.073	PDE6A,SLC26A2,TIGD6,HMGXB3,CSF1R,
**5**	150901111	151097281	86	0.063	FAT2,SPARC,
**5**	167755986	167946261	88	0.069	WWC1,RARS,
**5**	169793712	169992533	101	0.079	KCNIP1,KCNMB1,
**5**	171230798	171419658	36	0.068	FBXW11,
**5**	174481433	174678058	87	0.082	
**6**	13209794	13400667	73	0.066	PHACTR1,LOC100130357,TBC1D7,GFOD1,
**6**	39731430	39929194	75	0.064	DAAM2,MOCS1,
**6**	67730693	67929389	40	0.069	

**6**	69802200	70001186	32	0.071	BAI3,
**6**	114165133	114364702	55	0.066	MARCKS,LOC285758,FLJ34503,HDAC2,
**6**	147822074	148021909	88	0.074	SAMD5,
**7**	15021223	15215277	75	0.065	
**7**	19734101	19916190	48	0.069	TWISTNB,MIR3146,TMEM196,
**7**	95516569	95716383	55	0.069	DYNC1I1,
**7**	101697980	101895864	31	0.064	CUX1,
**8**	1778697	1978115	129	0.071	ARHGEF10,KBTBD11,
**8**	1983582	2181540	170	0.064	MYOM2,
**8**	16774970	16973443	66	0.070	FGF20,MICU3,
**8**	17780036	17977650	91	0.069	PCM1,ASAH1,
**8**	19204945	19401179	70	0.071	SH2D4A,CSGALNACT1,
**8**	26724326	26923058	75	0.065	
**8**	62370511	62566588	47	0.065	CLVS1,ASPH,
**8**	62803036	62969408	40	0.063	
**8**	63635874	63830366	46	0.064	NKAIN3,
**8**	64448090	64642614	32	0.068	
**8**	75207653	75405213	42	0.083	JPH1,GDAP1,
**9**	853004	1052722	77	0.064	DMRT1,DMRT3,DMRT2,
**9**	2468858	2668187	132	0.065	FLJ35024,VLDLR,
**9**	96792054	96990968	39	0.069	PTPDC1,MIRLET7A1,MIRLET7F1,MIRLET7DHG,MIRLET7D,
**9**	98670456	98867813	47	0.076	ERCC6L2,LINC00092,LOC158435,
**9**	111890197	112089520	65	0.064	FRRS1L,EPB41L4B,
**9**	133250275	133442778	73	0.080	ASS1,
**10**	6657775	6857219	101	0.087	LINC00707,
**10**	14574817	14774103	114	0.065	FAM107B,

**10**	21907016	22098701	18	0.066	MLLT10,DNAJC1,
**10**	28993801	29183994	79	0.069	LINC00837,C10orf126,
**10**	68813983	69013466	48	0.070	CTNNA3,LRRTM3,
**10**	70882515	71082214	67	0.073	VPS26A,SUPV3L1,HKDC1,HK1,
**10**	99443975	99642731	68	0.077	AVPI1,MARVELD1,ZFYVE27,SFRP5,GOLGA7B,CRTAC1,
**10**	112799928	112996845	69	0.077	ADRA2A,
**10**	132822263	133020132	116	0.066	TCERG1L,
**10**	133227554	133377136	70	0.073	
**10**	134045675	134244680	58	0.073	STK32C,LRRC27,PWWP2B,
**11**	57731052	57930210	51	0.070	OR9Q1,OR6Q1,OR9I1,
**11**	71239086	71419408	27	0.064	KRTAP5,KRTAP5,KRTAP5,KRTAP5,KRTAP5,
**11**	85332482	85526399	41	0.074	DLG2,TMEM126B,TMEM126A,CREBZF,CCDC89,SYTL2,
**11**	111037249	111233450	61	0.079	C11orf53,C11orf92,C11orf93,MIR4491,POU2AF1,
**12**	13927062	14125734	78	0.079	GRIN2B,
**12**	45975915	46145212	36	0.064	LINC00938,ARID2,
**12**	62272569	62466901	73	0.068	FAM19A2,
**12**	96309877	96491750	75	0.064	CCDC38,AMDHD1,HAL,LTA4H,
**12**	115616511	115816442	81	0.085	
**12**	118885039	119083566	54	0.086	
**12**	129547345	129745507	129	0.082	TMEM132D,
**12**	129955359	130151190	101	0.065	TMEM132D,
**13**	29841357	30036352	88	0.072	MTUS2,
**13**	96360079	96558852	24	0.067	DNAJC3,UGGT2,
**13**	96590060	96777428	19	0.085	UGGT2,HS6ST3,

**14**	22433866	22633640	90	0.065	
**14**	26284540	26484040	52	0.068	
**14**	30395066	30593866	48	0.084	PRKD1,
**14**	95358513	95556747	96	0.070	DICER1,
**14**	97650049	97849962	46	0.062	
**14**	101743391	101942768	63	0.081	
**15**	86840217	87037559	70	0.069	AGBL1,AGBL1,
**15**	87045550	87232413	68	0.066	AGBL1,
**15**	93939485	94135579	108	0.077	
**16**	4124300	4318455	58	0.070	ADCY9,SRL,LOC100507501,TFAP4,
**16**	19421858	19620862	60	0.069	TMC5,GDE1,CCP110,C16orf62,
**16**	79016116	79214272	133	0.073	WWOX,
**17**	9802584	9999784	86	0.065	RCVRN,GAS7,
**17**	51613235	51802835	39	0.063	
**17**	72929391	73128586	64	0.069	OTOP2,OTOP3,HID1,CDR2L,ICT1,ATP5H,KCTD2,SLC16A5,ARMC7,NT5C,
**18**	13271319	13471199	60	0.088	LDLRAD4,MIR5190,
**18**	47730011	47926178	58	0.063	CCDC11,MBD1,CXXC1,SKA1,
**18**	77839701	78015180	32	0.065	ADNP2,PARD6G,PARD6G,
**19**	47073819	47273268	43	0.069	PPP5D1,CALM3,PTGIR,GNG8,DACT3,DACT3,PRKD2,MIR320E,STRN4,FKRP,
**20**	18438954	18625194	33	0.077	DZANK1,POLR3F,MIR3192,RBBP9,SEC23B,LINC00493,DTD1,
**20**	40365781	40564041	52	0.079	
**20**	49960636	50160035	71	0.080	NFATC2,MIR3194,
**21**	17275170	17472366	45	0.066	
**21**	26476184	26669398	38	0.067	
**21**	37137766	37327654	31	0.073	
**21**	37338018	37536408	77	0.066	SETD4,LOC100133286,CBR1,CBR3,CBR3,

**22**	23815760	24004787	56	0.063	IGLL1,C22orf43,GUSBP11,
**22**	49484533	49683905	142	0.094	

Figure S1. PCA of our study samples with selected Hapmap 3 populations.

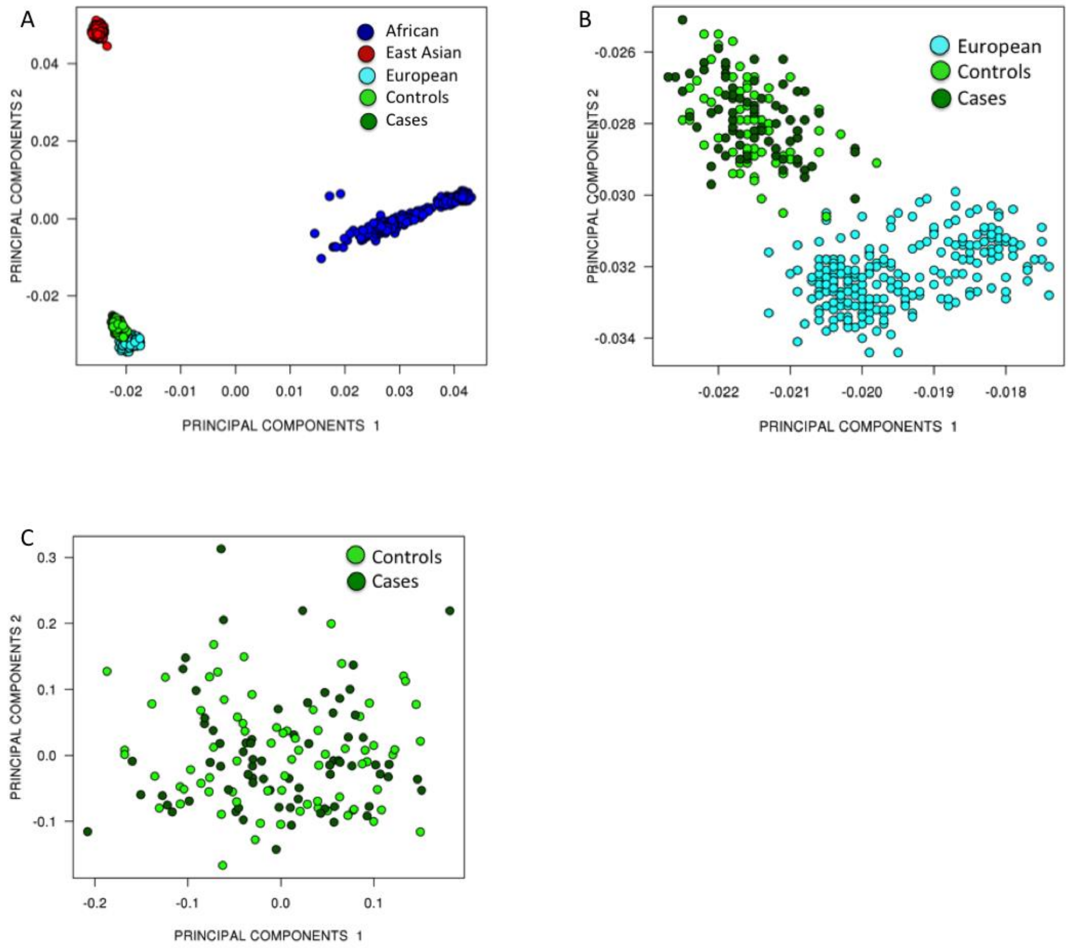

Populations in PCA analyses are from Hapmap3 and our cases and controls. For East Asian populations, we included all samples from CHB, CHD and JPT. European populations include all samples from CEU and TSI. African populations include all four African populations ASW, LWK, MKK, YRI. The PCA analysis is based on 49303 SNPs that are randomly chosen from the genome-wide SNPs. A) PCA of our study samples with selected Hapmap3 populations B) PCA of our study samples with Hapmap3 European populations C) PCA of cases and controls.
